# Integration of knowledge and local wisdom for disaster resilience in Anak Krakatau volcano

**DOI:** 10.4102/jamba.v15i1.1457

**Published:** 2023-06-28

**Authors:** Azhar Firdaus, Fatma Lestari, Suraya A. Afiff, Herdis Herdiansyah

**Affiliations:** 1 School of Environmental Science, Universitas Indonesia, Central Jakarta, Indonesia; 2 Department of Occupational Health and Safety, Faculty of Public Health, Universitas Indonesia, Depok, Indonesia; 3 Disaster Risk Reduction Centre, Universitas Indonesia, Depok, Indonesia; 4 Faculty of Social and Political Science, Universitas Indonesia, Depok, Indonesia

**Keywords:** disaster resilience, knowledge, local wisdom, Anak Krakatau volcano, tsunami

## Abstract

Indonesia lies on the Pacific Ring of Fire, where most of the area is highly unstable, making it a site of numerous volcanoes and earthquakes, so it needs to enhance its social system through knowledge, awareness, and local wisdom to create disaster resilience. Previous research has discussed resilience through societal knowledge and awareness, but there is a gap in understanding the local wisdom. Therefore, this study aims to present how the resilience process concerns with the community based on the local wisdom and knowledge for the community in Anak Krakatau, Banten. This research employs observations on the conditions of facilities and infrastructure of access road, in-depth interviews with local people, and bibliometric review over the last 17 years. A total of 16 articles were selected and reviewed for this study, after comprehensive analysis from 2000 documents. It is stated that preparing for natural hazards requires the integration of knowledge and local knowledge. Prior to the occurrence of a natural calamity, knowledge is dependent on the structure of a home in order to create a resilient structure, while local lore relies on the omens of nature.

**Contribution:**

The integration of knowledge and local wisdom can fulfil the resilience process in terms of preparedness and after effect of natural hazards. These integrations must be evaluated for disaster mitigation policies in order to develop and implement a comprehensive disaster mitigation plan for the community.

## Introduction

Over the last 10 years, about 3570 natural hazards have affected more than 134 million people in the world (International Federation of Red Cross [IFRC] 2018). These disasters have had a significant negative impact both locally and internationally on the socioeconomic environment (Hamza 2015; Monllor & Murphy 2017). In 2018, over 500 000 people were affected by 2573 natural hazards, of which 4800 people were killed and disappeared (The Indonesian National Disaster Management Agency website [BNPB]). Natural hazards are also at an all-time high in Indonesia, owing to the geographical conditions of the equator and the ring of fire, as well as the interconnection of the three tectonic plates (Astuti, Werdhiana & Wahyono 2021:101992).

Besides the tectonic plates, Indonesia has a potential threat from volcano eruption located in the islands of Sumatra, Java, Maluku, and Sulawesi. According to Indonesian history, there have been 10 large explosions in volcanoes that have impacted the community and cost life in Indonesia. The first was Mount Tambora, which erupted in 1815, killing over 80 000 people, and the second was Mount Krakatau, which erupted in 1883, killing 36 000 people (Pratomo & Abdurachman 2004:56–60).

Volcanic eruptions in Indonesia related to the caldera formation occurred on Mount Krakatau in 1883. This eruption affected the world climate as revealed by a decrease in temperature and also caused geological hazards in several parts of the earth (Kartadinata et al. 1997; Lipman 1997:198–218; Newhall & Dzurisin 1988; Sigurdsson & Carey 1989:243–270; Simkin & Fiske 1983:244–254; Sutawidjaja et al. 2005). The eruption of Mount Krakatau in 1883 threw more than 10 km³ of pyroclastic material into the air, both in the form of eruption hot clouds and eruptive ash. This eruption killed over 36 000 people because of tsunami waves caused by the collapse of the volcanic crater wall (sector failure) and the flow of hot eruption clouds into the sea (Camus & Vincent 1983:167-173); Simkin & Fiske 1983:244–254; Valentine 2000:571–580).

The first indication of the Mount Anak Krakatau embryo’s existence occurred from 29 December 1927 to 05 January 1928, as an underwater volcano at a depth of 28 m below sea level (Alcántara-Ayala 2002:107–124; Simkin & Fiske 1983:244–254). This mountain grows as a volcanic island (Mount Anak Krakatau), which until now in 2020, has reached an altitude of about 230 m above sea level. Approximately 4 years ago, in mid-December 2018, Mount Anak Krakatau increased its eruptive activity, resulting in a silent tsunami wave caused by an underwater landslide following the massive eruption of Mount Anak Krakatau on 22 December 2018, which is located in Pandeglang Regency, Banten Province.

Based on this historical background, it can be concluded that the coast’s condition around the Mount Anak Krakatau area, especially in the coastal area of the Pandeglang regency, Banten province has a significant impact on the community. The significant impacts that can be experienced for these coastal communities because of the eruption of Mount Anak Krakatau in the future are the loss of buildings and houses, loss of livelihoods, and deteriorating health conditions as a result of volcanic ash, as well as the psychological condition of the community.

Indonesia is home to a large number of indigenous people, whose customs and beliefs influence its cultural practices. Researchers and practitioners in Indonesia have recently shown a rising interest in examining these cultural practices that are connected to people’s knowledge and local wisdom around natural catastrophes. The question of whether this knowledge and local wisdom could improve policy mitigation in order to lessen the impact of natural hazards in the near future is what motivates various studies to explore how people use their culture practices (norms, customs, and local wisdom) to deal with natural hazards. In addition, these studies have shown how important it is for researchers and practitioners to look at the many knowledge systems, beliefs, and practices that local people have, as well as how these could be useful to those who plan development interventions (Bankoff 2003; Inan, Beydoun & Pradhan 2018:711–721; Mercer & Kelman 2010; Wilkinson 2018:459–474).

This development is seen in the policy of wide architecture from the humanitarian sector, which increasingly realises that it is important to put a people and community as an object of the impact in the natural hazards (United Nations 2015). There is more room for structural determinants of larger disaster-prone areas, such as cultural considerations or community wisdom, by shifting the emphasis from short-term disaster management to long-term disaster risk management based on the Sendai Framework for Disaster Risk Reduction 2015–2030 (World Humanitarian Summit 2015). The Sundanese people in Pandeglang Regency, Banten which was the location of the research, have had three views about the natural environment (Indrawardana 2012:1–8), namely:

- Viewing the natural environment as something to be respected, maintained, and cared for rather than something to be subdued. In essence, Sundanese attitudes and behaviour towards nature are more adaptable to the natural environment.
- The Sundanese people’s commitment to ecological harmony has a direct or indirect impact on how nature and their surroundings shape their character. This is typically made clear in proverbs, parables, or folktales that are full of life lessons and names for characters that borrow many phrases from nature.
- The natural environment for the Sundanese people is seen as an economic aspect to meet their daily needs. However, it is often used as a symbol for human life, ethics, and aesthetics in terms of nature. Nature is used as a supposition, metaphor for human character and behaviour, through expressions in comparative language, *kias*, or metaphors. Based on these languages’ form, the richness of flora and fauna in the natural environment of the Sundanese people can be seen.

Local communities’ involvement, which is the focus of this research, is fundamental and essential for building a sustainable early warning system to increase social resilience capacity (Mohanty et al. 2019:5–15). One of the involvements is about knowledge in the community regarding building materials of the houses to be resilient with volcano eruption. Previous research states that the knowledge regarding building materials in Baliau, Papua New Guinea (Mercer & Kelman 2010) uses traditional bush materials to build their houses. These materials are easily accessible, easily replaceable, and simple to erect and dismantle. The houses have traditional long sloping roofs in order to eliminate the potential for collapse under the heavy weight of volcanic ash in case of an eruption. There is the potential for fire, but the roofs are designed so that volcanic ash slips from the roofs to the ground, away from house walls, thereby significantly reducing the fire risk.

Another explanation for knowledge in Songhe, China, is invisible local knowledge (Lin & Chang 2020:101339). Local wisdom, known as ‘invisible local knowledge’, was created to address daily requirements by utilising available resources from the natural world and interpersonal connections. The use of social resources, natural resources, and disaster experience are the three categories that can be used to classify invisible local knowledge. In ordinary times, the people of Songhe constructed stone ridges to safeguard and utilise their land resources. This method reduced the risk of rockfall and debris collapse, maintained soil and water, decreased soil loss, boosted water discharge, and preserved the natural environment.

Still in China, and particularly in Xinjiang, communities practise disaster resilience and adaptation based on their previous experience in traditional rural settlements (Sun et al. 2022). For these settlements to continuously improve resilience, it is crucial to build water-conservancy infrastructure, with institutional guarantees provided by government-guided deployment. In addition, the knowledge gained from local construction experience with catastrophe adaptation might boost rural resilience even more. Finally, cultural and religious norms can give locals a behavioural foundation for planning and successfully adjusting to disasters. Similar procedures relating the experience and adaptability of natural hazards are present in Palu City, Indonesia (Yulianto et al. 2022). According to the findings, the community adaptation of the residents of Palu City was significantly influenced by their knowledge of and exposure to a phenomenon, the community’s characteristics, and the accessibility of local resources.

Firstly, the explanation of knowledge is provided by Bayan (Wahyuningtyas et al. 2019:1227–1235) who recognises warning signs of impending disasters, such as birds’ sounds and a yellow moon’s appearance. This knowledge explains that if the moon has turned red, there will be a disaster soon. This region (Pandeglang regency) is prone to earthquakes and volcanic eruptions, according to the Bayan Health Centre’s Bayan Disaster Knowledge. This statement explains that while providing calamity forecasting, individuals must contact the local office of Regional Calamity Management Agency (BPBD). This assertion contributed to the values, decision-making, and group cohesion.

Secondly, in addition to knowledge, there are beliefs widely held that superhuman entities such as ancestral and guardian spirits exist. Based on those beliefs from ancestral and guardian spirits, we will make a decisions from the traditional leader [*Pembekel*] via *Kiai* for information on how to live [*awik-awik gubuk*] and disaster. Thirdly is group solidarity with a strong cooperative culture. Every night, the tradition of community gatherings is carried out. Meetings are customarily held during formal events and Islamic holidays. In anticipation of the disaster, citizens are communicating openly.

Another example that has some values in their culture is in Sabuk Janur Mount Lawu (Lestari et al. 2018). These values form some public awareness to protect the environment and overcome natural hazards. However, these values only have an important role in conserving farming management. Another article (Kangabam, Panda & Kangabam 2012:1632–1642) proved that, in the event of a potential disaster with landslides and earthquakes, more education was provided with local or community conditions and residents of the community. So, the community and the residents could understand what to do if natural hazards happen. On the contrary, in other cases, the level of knowledge in volcanic hazards in Japan (Kuri & Suppasri 2018:1082–1095) has not been associated with disaster awareness or evacuation awareness.

Another example has happened in Mount Merapi with some resilience processes are taking place as part of disaster preparedness (Pawirodikromo 2018), such as the prevention phase, early warning phase, evacuation phase, care and discipline, response phase (public relationship), and capacity building of craftsmen, buffer boundary, and comprehensive contingency planning. With such a great disaster preparedness in Mount Merapi, the people of Karo in the Mount Sinabung completed the resilience process with sense of belonging (Mori et al. 2019:290–303). This sense of belonging makes the people of Karo want to adapt and survive after the eruption. Also, these people are willing and responsible to make certain modifications of the certain aspects of their culture that do not violate its core values. Sinabung volcano also has some mitigation and adaptation policies, which are as follows:

- Relocating to identify, assess, monitor, and implement an early warning system.
- Using knowledge, innovation, and education to build a culture of safety and resilience at all levels.
- Making ‘Disaster Risk Reduction or DRR’ a national and regional priority implemented through strong institutions.
- Reducing the factors underlying the causes of the occurrence or increase of the risk of disasters.

Based on the previous statement regarding the history of natural hazard and about local wisdom to mitigate with this, there is a gap that will be addressed in the research, such as:

- A lack of information and technology with the early warning system in coastal area in Pandeglang regency, Banten Province.
- A lack of knowledge and education on how to build a culture of safety and resilience with the communities.

This article will demonstrate the integration of knowledge and local wisdom from the communities based on the high potential for disaster in the Anak Krakatau volcano to address the two research gaps previously mentioned. The resilience procedure will be implemented as part of the community’s disaster risk management strategy. The purpose of integration is to create a harmonious relationship within the environment, which will save not only the inhabitants but also the environment.

## Methods

The research method is a qualitative approach to elicit detailed information about the community’s awareness of the catastrophe resilience of the Anak Krakatau volcanic eruption. This study employs observations of the facilities and infrastructure of the access road, as well as in-depth interviews with locals in the Sumur subdistrict of the Pandeglang regency, which is located close to the Anak Krakatau volcano. In addition to completing the data from observations and in-depth interviews, research is doing a systematic literature review in term of community and local wisdom to better comprehend the Anak Krakatau volcano eruption’s resilience. Details of data collection methods are given below.

## Observation and in-depth interviews

It is vital to plan so that the road is more accessible for evacuation of the potential victims. This can decrease the number of disaster victims and enhance public awareness to be more vigilant in dealing with catastrophes. Locals are interviewed in-depth about the 2018 tsunami calamity and their knowledge of tsunami-resistant building design as well as observation of the road access in the area and the infrastructure that supports it.

## Systematic literature review

Secondary data are gathered via a thorough review of the literature. A systematic literature review uses the appropriate research to find and critically evaluate as well as collect and analyse the research’s data (Liberati et al. 2009:e1–e34). The VOS Viewer software was used for data processing and literature reviews. With the help of this software, bibliometric networks made up of journals, researchers, or specific publications may be built and visualised. Based on citation, bibliographic coupling, co-citation, or co-authorship relationships, these networks can be built (Van Eck & Waltman 2022).

## Data extraction and analysis

### Data extraction

Data extraction from the observation is captured by photograph and video to analyse the current condition regarding road access for the evacuation route. Otherwise, with the in-depth interview the research has a guidebook to interview and provides a recording tool to record the whole interview process. Data from the photograph, video and sound recording will be transcribed and descriptively analysed.

Data extraction with secondary data will focus on natural catastrophes, volcanic effect, and local knowledge to promote natural hazard mitigation. This list of keywords relates to the purpose of the research, which is to address the information and technology associated with the early warning system as part of disaster mitigation, as well as the knowledge and education that will foster a culture of safety and resilience in communities. Also, these keywords were selected because it relates to the subject of the study, which is tsunamis caused by volcanic activity. To accomplish this, a thorough literature search was conducted on the following electronic database with Scopus database using VOS Viewer, which yielded 2000 documents published within the past 17 years. The first step is to search within the keywords, natural disasters, and resilience, to see the interconnection between the other keywords.

**FIGURE 1:**
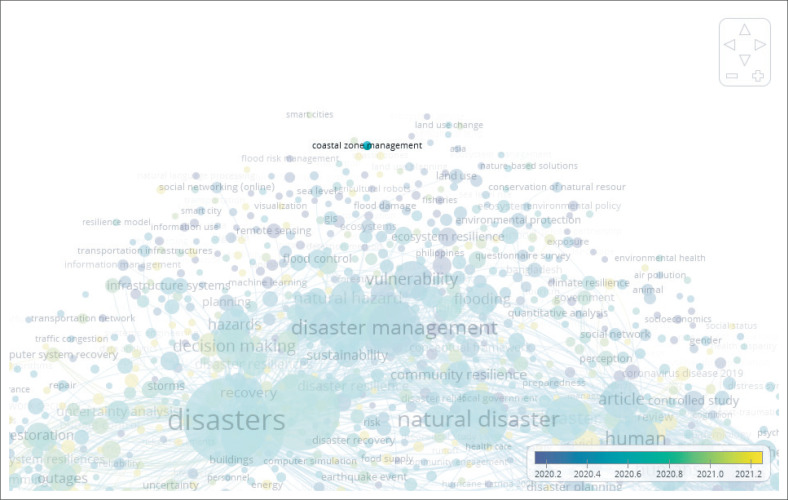
Overlay visualisation in terms of coastal zone management.

**FIGURE 2:**
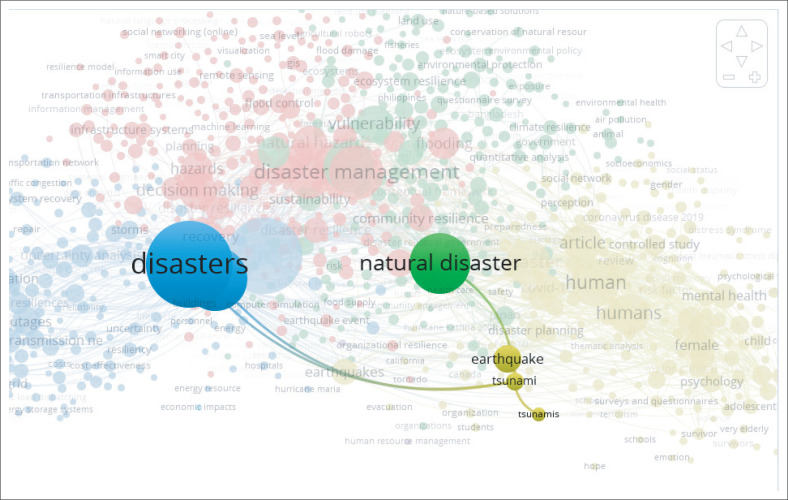
Network visualisation in terms of Tsunami subject.

Based on the VOS Viewers results from 2000 documents with the keywords natural disasters and resilience (Figure 1 and Figure 2), there are two subjects that require more extensive treatment: administration of coastal areas and tsunamis. Also, the result shows that there is more to explore for the resilience process that focuses on coastal management, volcano, and role of the community. During the examination based on 2000 documents, there are 88 articles that potential was identified over the last 17 years since 2006–2022. The remaining articles were retrieved and reduced to 16 articles throughout the assessment in terms of title, abstract and main findings of the articles.

### Data analysis

The extracted data from the observatory and in-depth interviews were analysed using the Miles and Huberman model. These models are divided into three phases. First phase does data reduction or simplifies the data. The collected data are selected based on their relevance to the research’s objectives as a second phase. The third phase of the data analysis concludes with the presentation of the appropriate model of the Anak Krakatau volcano’s resilience process.

The analysis of the literature review started with the identification of common main results from the selected studies. The findings were broken down into descriptive themes. The 16 papers’ geographical precinct is not limited to a single region but covers the Papua New Guinea, United States, Taiwan, and Indonesia.

### Ethical considerations

The research provided in this article, including observation, in-depth interview and literature review has been approved by the University of Pembangunan Nasional Veteran Jakarta with ethical approval number: 384/IX/2022/KEPK on 13 September 2022, in Jakarta.

## Results

The following are topics within issues in the research location; information and technology with the early warning system, and knowledge and education to foster a culture of safety and resilience. These topics will be discussed on knowledge and local wisdom to enhance the natural hazard mitigation process. In addition, by examining the studies and combining them with the observation study objectives, an in-depth interview, and a systematic literature review were conducted. The following subsection goes into further information about each of these.

### Issues in Pandeglang regency, Banten province, Indonesia

The Pandeglang regency is a district in the western portion of the island of Java. Geologically, this region has a very complex tectonic environment, as it is situated between various oceanic plates and actively moving continental plates. In this region, three tectonic continents converge: the Indo-Australian Plate, the Eurasian Plate, and the Pacific Plate. This region is prone to collisions between the Eurasian continental plate, the Indo-Australian oceanic plate, and the Pacific oceanic plate because of the convergence of these three plates. Where the collision occurred, the oceanic plate subducts beneath the Eurasian continental plate, formed a subduction zone. Along the subduction zone of the continental plate, a chain of volcanic islands or a magmatic belt form formed, extending from Sumatra and southern Java to Nusa Tenggara and Banda. It is called the Sunda-Banda magmatic arc (Hamilton 1988:1503–1527).

Nearly every year, the Pandeglang regency is struck by natural calamities including landslides, floods, tornadoes, and the threat of a tsunami. According to the Pandeglang Regional Disaster Management Agency, the Pandeglang district is a national disaster-prone area and is one of the 20 national disaster-prone areas. The threat of tsunami caused from Mount Anak Krakatau is the focus of this article.

### Information and technology with the early warning system in the coastal area

This research focuses on the Sumur district, Pandeglang regency, Banten province coastline. Because this location was greatly affected by a tsunami when the Anak Krakatau erupted. It was proved in mid-December 2018, when Mount Anak Krakatau showed an increase in eruptive activity, which resulted in a silent tsunami wave because of an underwater landslide following a massive eruption on 22 December 2018. There are seven sub-districts in Sumur district and five sub-districts were affected because of silent tsunami. The maximum impact for these tsunamis is on Sumberjaya and Kertajaya sub-district.

Based on this tragedy, previously there was no siren-based early warning system to warn of abrupt high waves along the coast. It is essential to have a siren in coastal areas prone to earthquakes and volcanoes that cause tsunamis. These sirens will enhance the speed with which communities are evacuated to higher ground. The informants who were interviewed in Sumberjaya sub-district stated:

In Bahasa:

… *Tidak ada pak Sirene atau informasi apa saja dari pemerintah setempat. Kami (warga) hanya mendengar orang-orang teriak ‘Cah Laut! Cah Laut’. Kami langsung terburu-buru lari ke tempat tinggi …*

In English:

[… No sirens or any information from the local government. We (residents) only heard people shouting ‘High Wave! High Wave’. We immediately rushed to the high ground …] (Interviewee Mr Endin, male, Sumberjaya).

Also, in Ujungjaya sub-district, there was a lack of early warning system when the tsunami hit in 2018. As a result there was misinformation about the community’s response when the disaster hit and increased loss of life and property damage along the coastal area:

In Bahasa:

… *Saya dan teman-teman ketika tsunami lagi mancing Pak. Tiba-tiba saya melihat gelombang tinggi yang menyapu saya dan teman-teman di perahu. Sebelumnya saya tidak ada informasi dari telepon atau sirene di lokasi saya mancing* …

In English:

[… When the tsunami hit, my friends and I were fishing, sir. Suddenly I saw a high wave that swept me and my friends in the boat. Previously, I had no information from the phone or sirens at my fishing location …] (Interviewee Mr Dadin, male, Ujungjaya).

**FIGURE 3:**
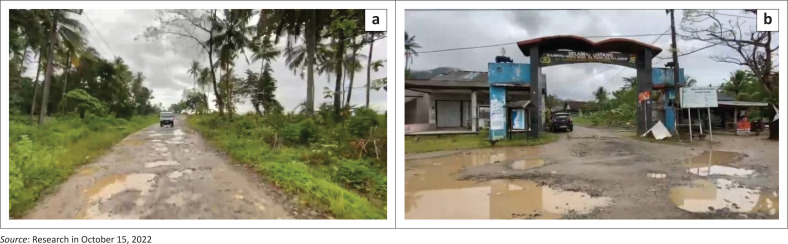
Some of the access roads in Tamanjaya village, near the Pani’is beach, Sumur district, Pandeglang regency, Banten province, Indonesia.

Besides the early warning system, there is an urgent need for the government to provide building materials and practices that are environmentally friendly and resilient to earthquake and tsunamis all along coastal areas in Pandeglang regency. Also, more education and knowledge should be provided regarding how to make an evacuation if there is a potential disaster, especially with the tsunami from Anak Krakatau volcano. Because, after some observation in the sub-districts of the location, the access road is still damaged. These damages are in the vulnerable area (coastal area) with the tsunami from Anak Krakatau volcano and it will be difficult for people (especially) to escape for evacuation. This condition is presented in Figure 3.

Aside from a lack of information and road access along the coast, there is a lack of technology that has not been fully implemented in the coastal area of Pandeglang regency, Banten province. In the coastal area, especially in Sumur district, there are frequent power outages resulting in the telephone network also shutting down. It happened when the research was conducted on 15 October 2022, during night the electricity was turned off and there was no telephone network. Based on this, it needs more essential technology facilities, such as handheld transceivers and generators to provide more information, especially with natural hazards because of Mount Anak Krakatau. Because when tsunami happened in 2018, there was no way of informing citizens to get out.

### Knowledge and education to foster a culture of safety and resilience in communities

Knowledge on natural hazards exists in the Baduy community in Pandeglang regency, Banten province, Indonesia. They have knowledge, especially on construction buildings. The construction buildings have simple technology but upholds the wisdom of the environment (Permana, Nasution & Gunawijaya et al. 2011:67–76). Baduy houses are simple stilt houses made of wood, bamboo, palm fibre, and thatch; they all have the same shape and design. Baduy houses on stilts are generally associated with the belief that the house, as the focal point, represents a neutral power that exists between the underworld and the world above. The house should not be built directly touching the ground (as part of the underworld). In particular, the Baduy house based on its vertical arrangement reflects the division of the universe (Permana, Nasution & Gunawijaya 2016). The legs or pillars symbolise the underworld (the world of darkness, hell); the body or walls and space in them represent the middle world (the world of the living universe), and the head or roof symbolises the world above (the eternal world, heaven).

**FIGURE 4:**
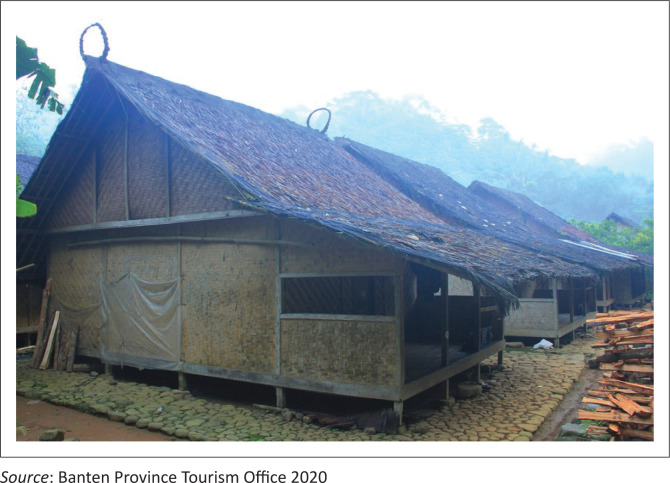
Baduy traditional houses.

The Baduy community’s local wisdom in traditional building traditions related to earthquake disaster mitigation can be found in construction, building connection and tie techniques and the use of *umpak* (Suparmini, Setyawati & Sumunar 2014:47–64). Houses are built using materials derived from their surroundings in their livelihood, such as wood and bamboo. The building structure is built on a frame system made of wood in the form of rectangular beams and pillars. The structure of the wall covering is made of woven bamboo chamber [*geribig*], which is preserved in its original colour and character. The houses of Baduy communities can be seen in Figure 4.

The split bamboo is also used to form the covering structure at the end of the woven bamboo. All construction details are completed with the principles of ties, supports, pegs, interlocking supports and hooked joints. The Tangtu Baduy are prohibited from using nails in the process of making houses, and are replaced with rattan and bamboo, or with the peg technique. The structure of the floor of the house is generally used bamboo, which is made in the form of sheets called *palupuh*. In addition, for the main *hateup* structure [roof], thatch roof [*kiray*] is used with bamboo and rattan as a binder. So that if in the future an earthquake occurs, the structure of the house made with bamboo and rattan will move dynamically to avoid damage or destruction.

The construction of structures in Baduy communities is governed by customary or *pikukuh* building regulations and prohibitions. These rules for building construction have been inherited so that houses do not collapse readily (Suparmini et al. 2014:47–64). This method is effective for disaster mitigation, and it has been documented that the Baduy tribe’s environment is rarely damaged.

Besides in Baduy regarding traditional knowledge for construction buildings, in Lombok, after the 2018 earthquake, there are research that proposed building construction design called Bamboo incremental house (Kusuma 2022:119–127). This study suggests a disaster-resistant incremental housing design made of bamboo that may be tailored to the needs of the inhabitants and support their economic activities after recent natural hazards. These architectural options are not only cost-effective but also encourage sustainable building practices throughout the society.

Regarding the resilient process in Mount Anak Krakatau volcano, the people in the Pandeglang regency are knowledgeable about the construction that are resilient to the earthquake and tsunami that occurred. However, the people are having some challenges with two things, namely insufficient funds and job opportunities. In terms of insufficient funds, the communities in coastal area, especially in Pandeglang regency, know that the building materials from wood, bamboo, palm fibre, and thatch are resilient to natural hazards (earthquake and tsunami). However, these building materials in Pandeglang regency, are hard to find and cost highly to collect, compared with the current condition in Lombok (Kusuma 2022:119–127). So, the communities move to bricks, concrete, or any similar building materials that are cheaper. From the interviews, one of the Village chiefs in Sumberjaya, indicated that:

In Bahasa:

… *Kita tahu bahan tahan gempa dan tsunami itu dari kayu dan bambu. Tapi bahan-bahan itu semua mahal, jadi kita bangun lagi dengan batu bata, beton, karena bahan-bahan itu lebih lebih murah …*

In English:

[… We are aware that wood and bamboo are earthquake- and tsunami-resistant building materials. However, it was expensive, so we rebuilt the house from masonry and concrete because it was more affordable …] (Interviewee, Mr Endin, male, Sumberjaya).

To be well-known and comprehensible regarding the knowledge of building construction to the community, direct practical experience is required to build the structure and test it if there is an earthquake in the area. For the construction of wooden and bamboo structures, direct practice is used. The principles of ties, supports, pegs, interlocking supports, and hooked connections are utilised to complete all of these construction details. After the structure is built, it will be simulated if an earthquake of magnitude greater than 6.0 on the Richter scale occurs, as well as the community’s response. The communities must prepare with disaster preparedness bags before a disaster strike for each family. This bag or suitcase contains essential documents such as deeds, vehicle registrations, diplomas, birth certificates, among others. Clothing must also be included in the emergency supply kit. Multiple outfits should be prepared for 3 days: beginning with underwear, pants, outerwear, blankets, towels, and raincoats, among others.

The communities may react to the earthquake in panic, which is one of the potential difficulties that may arise with this knowledge. As a result of the panic response, communities neglect to pack their disaster preparedness bags with essential documents and supplies for evacuation. The solution is for communities to be prepared with this bag before the next calamity strikes.

In terms of job opportunities, communities in the coastal area have various jobs in the coastal area, such as fishing, small businesses, workshops, and food stalls. When the government suggests moving their jobs to the location more safer than coastal area, which is tsunami prone, the communities have some challenges. Because this is the only job that they do, they do not have any opportunities besides those jobs. So, they have already rebuilt the business buildings to start their economy, even though they still have trauma from the tsunami in 2018.

Besides the insufficient funds, community’s response, and job opportunities, the research also addressed the local wisdom in the coastal area. Most religious or traditional leaders will be obeyed by the community. The religious or traditional leaders will incorporate local wisdom within communities and use government policies aimed at mitigating natural disasters. For some, in the littoral region of the Pandeglang regency, there is no written or implemented local wisdom present. However, there were some natural indicators that occurred prior to the 2018 tsunami that were neither implied nor defined as local wisdom.

Firstly, the sky’s condition at sunset, just before the tsunami struck. The sky in the night before tsunami happened around 21:00–21:30, as bright and the moon was clearly seen. This phenomenon is similar to previous research indicating that when birds are heard and a yellow moon appears, and if the moon has turned red, a calamity is imminent (Wahyuningtyas et al. 2019:1227–1235). Secondly, the emergence of fish and shrimp to the surface. This phenomenon was witnessed in Sumberjaya village before tsunami struck in 2018. Some of the people saw this phenomenon, but they became confused, and wondered why the fish and shrimp were on the surface. In fact, shortly after this phenomenon, a massive tsunami between 5 and 10 m in height struck the coast and forced people to evacuate. If the communities could understand this phenomenon earlier, they would have more time for preparing to evacuate to higher place and to reduce the loss of victim in the communities.

Thirdly, the emergence of one crocodile in Pani’is beach, Tamanjaya village. Some people at dawn, before tsunami struck in 2018, saw the emergence of one crocodile in Pani’is beach. This crocodile went up and down to the sea and coastal area many times. Some people, even saw the crocodile standing up on two legs. A few hours after this phenomenon, a huge wave came and swept all the houses in the coastal area in Pani’is beach.

Before a natural disaster occurs, communities must be made more aware of these issues, which require a basic understanding of building construction and natural warning signs. A young and elders’ community should be established to make some policies regarding how to increase the awareness if the disaster happens. These policies should include how to construct the earthquake resistant buildings and how the communities should prepare, read the signs, and evacuate during natural hazards. Specific actions will be explained in more detail in discussion section.

## Discussion

This study focussed on the current condition and how to improve the coastal area regarding the potential threat of Anak Krakatau volcano based on observation in the study’s location, in-depth interview and previous studies within the last 17 years. This research observed the condition of infrastructure and houses; for in-depth interview, the researchers interviewed some of the survivors and people who know the condition of coastal area during natural hazard. In addition, for previous research conducted within the last 17 years, 2000 documents were reviewed and 16 were chosen based on keywords such as, natural disasters, volcanic impact, and local knowledge to improve natural hazard mitigation. Based on these methods of collecting data, the studies showed that the resilience process needs the integration of knowledge and local wisdom to make communities in coastal area resilient against earthquake and tsunami events caused by Mount Anak Krakatau.

Combining environmental justice, local knowledge, and post-disaster planning, the knowledge to be implemented in the littoral area of Pandeglang regency, Banten province, has been acknowledged in Baliau, Papua New Guinea, since 2010 (Mercer & Kelman 2010). Villagers in Baliau use traditional bush materials to build their houses. These materials are easily accessible, easily replaceable, and simple to erect and dismantle. Also, lessons learnt from the past are important to face a future threat with natural hazard (Mercer et al. 2007:245–256). Taji Village in Indonesia’s Malang regency’s Bromo Tengger Semeru (BTS) area has also adopted this historical lesson (Masruroh et al. 2022:775–781); it is prone to landslides and the communities are aware about it. Therefore, they have incorporated conservation efforts into local wisdom by planting multi-strata plants with woody plants and horticultural crops on each section of the slopes.

Besides, in the United States of America, particularly in the state of Washington in 2009, there is some traditional knowledge regarding tsunami emergency management (Becker et al. 2009). According to their findings, traditional infrastructure knowledge can be utilised effectively for natural hazard education and to improve warning response. This infrastructure knowledge will enhance earthquake and tsunami resistance. Later in 2011, there was some integration within the Christian tradition (knowledge) and hazard prevention policies (Chester & Duncan 2011:85–95). This integration is needed because the geographic location is vulnerable to volcanic eruptions and earthquakes. Also, the communities need to learn and mitigate the potential of natural hazards. This integration was deemed necessary to incorporate a religious element into government disaster mitigation policies. Some regions or communities with strong faith in God and a belief that all natural systems, including volcanoes, disasters, and ecosystems, are God’s will require a religious dimension. This religious aspect as a rule in local communities is intended to raise awareness of natural hazards.

The development in Papua New Guinea, Indonesia, and USA since 2007–2022 that has integration between traditional knowledge in terms of infrastructure and their beliefs of the natural disaster are God’s will. This integration has not been implemented fully in the coastal area in Pandeglang regency, Banten province, especially with the infrastructure and how communities bond with the nature as a God’s gift. The communities are well-known for the traditional knowledge in terms of infrastructure. Based on previous statement, these materials that use traditional knowledge are hard to find and are costly to collect, such as wood, bamboo, palm fibre and thatch. So, the communities move to bricks, concrete, or any similar building materials that are cheaper. In addition, communities are not accustomed to recognising the signs of nature if there are peculiar activities in comparison to other days. Therefore, the communities require accurate information regarding this and how to respond to this phenomenon.

In 2016, the Sundanese communities (Maknun & Busono 2016) who are located mostly in the western part of Java Island had some knowledge of how to build a house to protect from nature, such as rains, wind, sunshine, and animals. This research needs to be improved to explain how Sundanese communities build their houses to protect from the earthquake, tsunamis and volcano that has been implemented in Baliau, Papua New Guinea (Mercer & Kelman 2010). However, this knowledge will not be denied (in the future), to build the construction of houses or any other buildings that will be strong enough to face natural hazards. These structures are also intended to make communities safer and more resistant to tsunamis and volcanic eruptions.

Communities are encouraged to use traditional knowledge to construct earthquake- and tsunami-resistant infrastructure based on construction technique of Baduy communities and people in Papua New Guinea. It is proved that communities after the tsunami in 2018, have a lot of workshops, training and knowledge sharing regarding how to build a good infrastructure that is resilient with natural hazard, make an evacuation when disaster happen and how to make a preparation in one bag that included the letters and things that are important for each family (Disaster Preparedness Bag). However, the government policies must not only share this knowledge, but provide some affordable or free materials to build the houses and building. So, the communities will have a rapid recovery after the disaster.

Several reconstructions in 2018 use disaster information gleaned from the study of science, environment, technology, and society as a theme. To fulfil the knowledge necessary to mitigate natural hazards, it is necessary to acquire a lesson from past and inherited local wisdom (Atmojo et al. 2018:204–213). Local wisdom has been incorporated into this reconstruction. Its implementation process was able to reconstruct and increase the disaster management knowledge. Some local wisdom regarding the signs of natural hazards is shown in Lombok, Indonesia (Wahyuningtyas et al. 2019:1227–1235). There are some disaster mitigation methods that were shown in cultural tourism presented in 2019. Communities recognise warning signs of an impending disaster such as sound of birds and the appearance of yellow and red moon.

Based on the experiences that occur in Lombok it similarly happened in Sumur district. The communities recognize that the night sky is clear and that there were warning signs in the coastal area prior to the calamity, such as the sight of crocodiles on Pani’is beach and the surfacing of fish and crabs. However, these signs appeared before the tsunami struck in 2018 in Sumur districts, communities still do not know why this phenomenon happens. The technology to warn communities with a siren is not available in most coastal area in Sumur district. In future, it is necessary to build a comprehensive infrastructure regarding the siren, based on the increasing sea level.

In Aceh, Indonesia, there are some opportunities and challenges to enhanced community engagement in terms of early warning system in disaster risk reduction (Sufri et al. 2020:2691–2709). The communities have some challenges with the four elements of early warning systems in Aceh such as risk knowledge; monitoring and warning; dissemination and communication; and response capability. To improve this, as an opportunity, the communities need the role of respected figures such as traditional religious leaders, local customary and sharia practices. Using certain figures, practices, and written policies to illustrate how to raise awareness about natural hazards, will increase community engagement. Those challenges in Aceh also happened in coastal area in the Sumur district. There are few respected figures such as traditional religious leaders, local customary and sharia practices concern with mitigation of natural hazards. Currently, some small groups are running a programme to plant a mangrove along the coastal areas to prevent high waves that may occur at any time. This program will be comprehensive if infrastructure such as tsunami sirens and earthquake and tsunami-resistant homes are constructed.

Some of the local wisdom from traditional knowledge that are previously stated are needed to send to the government as an organisation who is responsible to implement the mitigation process with the natural hazards. This was done in a research in 2020 that was located in Taiwan (Lin & Chang 2020:101339). There are some metamorphoses from local wisdom to involuted disaster knowledge for disaster governance in Taiwan, especially from the landslide-prone tribal community. The local communities have invisible local wisdom to meet daily needs using natural environment resources and social relationship. This local wisdom needs to be integrated for disaster management, so the daily needs will be fulfilled; also, the communities should have some knowledge to prevent the potential disaster.

Disaster mitigation in Indonesian communities of Shifting Cultivators in 2021 is based on local wisdom (Hos, Roslan & Supiyah 2021:237–243). Local wisdom is a structure that has been kept up to manage the sustainability and support of forests. It illustrates how the Tolaki people have for many years avoided natural hazards through their *monda* practices. In addition, the laws and customs governing the opening, burning, and cleansing of land according to the *monda’u* tradition must be followed.

In Indonesia since 2022, three patterns or kinds of disaster mitigation based on conventional wisdom – belief, knowledge, and engineering are used, and the disaster learning process incorporates a variety of teaching strategies into each subject (Suarmika et al. 2022:102874). This reconstruction is learned in Indonesian elementary schools. The disaster mitigation process based on the local wisdom and the knowledge of rapid evacuation will be more effective if it is taught in elementary schools and well-coordinated from home. It is presented in the research in 2022 in terms of evacuation model to reduce the volcanic risk in Mount Merapi, Indonesia (Chasanah & Sakakibara 2022:8110).

Based on the data collected from observation, in-depth interviews and systematic literature review, there are some knowledge and local wisdom that must be implemented to be resilient in natural hazards, especially with Mount Anak Krakatau volcano. These two key points must be integrated into governance policies that will be implemented in young and elder communities. So far, governance policies to mitigate natural hazards only present how to prepare and survive technically with natural hazard. Inputting the knowledge and local wisdom to governance policies, will make the mitigation process more comprehensive and implemented to the community. The integration that needs to be realised are knowledge to build a strong and safe infrastructure and local wisdom to understand the sign from the nature and how to be prepared that will be optimised to make an evacuation when the disaster happened.

For infrastructure, the communities need to give an access evacuation route with clear route and meeting point. This means, the route of evacuation should be smooth with a certain meeting point. It should make it easier for elderly people to run to safer place when the disaster happens. And for local wisdom, the communities need early warning system in the coastal area (especially, coastal area in Pandeglang regency) with the sign of nature that should be combined with siren and the radio information from Handheld transmitter (HT). It is potential needs, because as a previously mentioned, when the disaster strikes the electricity is suddenly turn off and there are no services available for mobile phones.

## Conclusion

This study presents a comprehensive analysis of the research based on observation, in-depth interviews, and a systematic literature review in order to develop a resilience procedure that integrates knowledge and indigenous wisdom with the Anak Krakatau volcano. This integration will be incorporated into disaster mitigation policies in order to make them comprehensive and implement in the community. The study also found that there is a need for further research into a number of natural hazards (not just volcanic eruption) that have been extensively studied and correlate with knowledge and local wisdom.
